# Congenital Bleeding Disorders: Managing Central Nervous System Bleeding in an Adult Hemophiliac

**DOI:** 10.7759/cureus.36906

**Published:** 2023-03-30

**Authors:** Anurag Dahra, Zainab Mehdi, Monica Gupta, Vijendra Patle, Seema Sharan

**Affiliations:** 1 General Medicine, Government Medical College and Hospital Chandigarh, Chandigarh, IND; 2 General Medicine/Emergency Medicine, Government Medical College and Hospital Chandigarh, Chandigarh, IND; 3 Medicine, Government Medical College and Hospital Chandigarh, Chandigarh, IND

**Keywords:** recombinant factor viii, haemophilia, factor viii inhibitors, intracerebral haemorrhages, congenital bleeding disorders

## Abstract

Congenital bleeding disorders remain a challenge to healthcare in the developing world. Despite the initiation of gene therapy almost five decades ago, the natural history of hemophilia remains the same. The cost of concentrated plasma factors, the development of a high titer of inhibitors in severe hemophilia A (HA), and the associated enhanced propensity of ICH make advancements in disease management questionable. Severe cases of hemophilia die young due to spontaneous central nervous system bleeds due to the lack of standard guidelines for plasma concentrate replacement and the limited availability of products due to the associated economic burden. Monoclonal antibodies, although a promising option as a standardized prophylactic treatment, remain underutilized due to availability and accessibility issues.

Here, we report the case of a 28-year-old male with HA who presented to the emergency with a progressively worsening headache, nausea, and elevated blood pressure. He had an intracerebral hemorrhage (ICH) successfully managed with decongestants and factor VIII supplementation.

## Introduction

Congenital bleeding disorders (CBDs) rarely present with central nervous system bleeding (CNSBs) except for factor XII (FXII D) deficiency where CNSB is seen in early life in almost one-third of patients. Among others at risk are severe hemophilia A and B (HA and HB), factor VII deficiency (FVII D), factor X deficiency (FX D), factor V deficiency (FV D), factor II deficiency (FII D), and afibrinogenemia. Hemophilia A and B are X-linked recessive mutations with an incidence of 1/5000 and 1/30000 male births, respectively. Musculoskeletal bleeding constitutes 70-80% of total events followed by mucocutaneous bleeding, gastrointestinal bleeding, and CNSBs. Atraumatic spontaneous and recurrent bleeding is most commonly associated with severe forms of disease having a residual plasma factor level of <1%. The risk of CNSB is highest in the first few days of birth although uncommon in the neonatal period. Afterward, a hemophilic male is 20-50 times more likely to develop intracerebral hemorrhage (ICH) as compared to a non-hemophilic counterpart. A recent review based on studies done in the USA, France, India, and Germany estimates the risk to be variable between 290 and 796 per 100000 males [1-4]. Among the various identifiable factors for ICH in hemophilia are a severe form of the disease, certain specific genetic mutations, development of inhibitors against exogenous factors, prior ICH, infection with and treatment of hepatitis C or HIV, and hypertension [3,4].

Various measures have been proposed to prevent bleeding complications in hemophilia right from birth in confirmed carriers and populations with increased rates of consanguineous marriages. Opinions vary right from the choice of mode of delivery to assisted delivery methods and early replacement of factors. No form of delivery attenuates the risk of ICH and assistance in delivery puts the patient at increased risk, whereas early replacement is linked to early inhibitor development [2-5].

Here, we report the case of a 28-year-old male with severe HA who presented to the emergency with progressively worsening headache, nausea, and elevated blood pressure. He was diagnosed to have ICH successfully managed with decongestants and factor VIII supplementation.

## Case presentation

A 28-year-old male presented to emergency with a chief complaint of headache since a few hours prior to presentation. It started suddenly and was increasing in intensity but was limited to the bilateral frontotemporal region. It was associated with mild nausea but he did not complain of any visual changes, paresthesia, or paresis. There was no history of photophobia, vertigo, or abnormal body movements. The pain was exacerbated upon coughing and sneezing. He didn’t have similar episodes in past. The patient had over-the-counter (OTC) analgesics, which did not provide relief. He was a known case of severe HA since childhood and had been on on-demand replacement therapy. He had a history of prior spontaneous musculoskeletal bleeds with evidence of arthropathy in his right elbow and knee. He did not smoke and was an occasional alcohol user.

Upon primary assessment, in an emergency, his vitals were BP - 170/80 mmHg, pulse rate (PR) - 122/min, and afebrile with random blood sugar (RBS) - 98 mg/dl. He was conscious and oriented to time, place, and person, with intact higher mental functions and a Glasgow Coma Score (GCS) of 15/15. Tone, power, and reflexes were normal, with intact sensory perception. Plantar reflexes were bilateral flexor responses with both pupils being normal and reactive to light without any evidence of hypertensive retinopathy or papilledema on fundoscopy.

**Figure 1:**
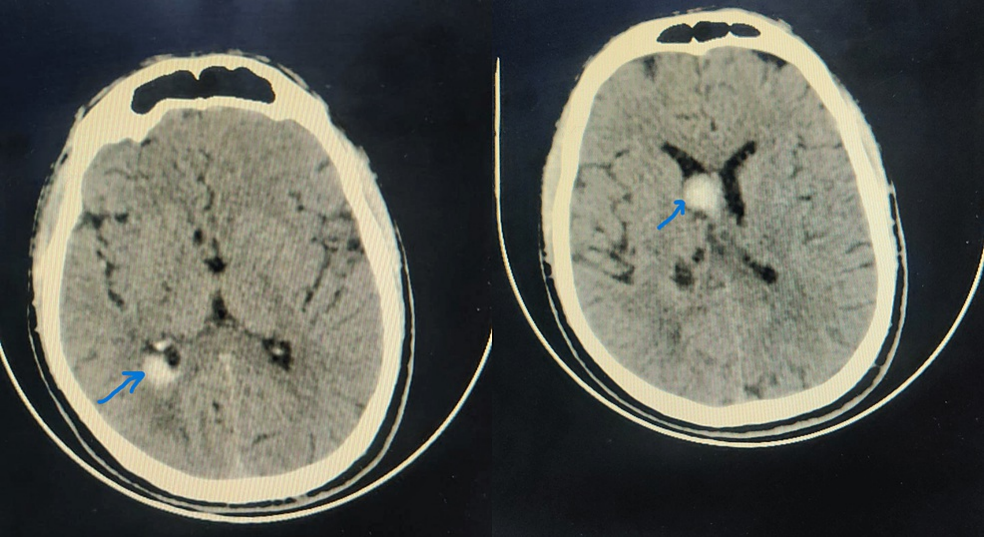
NCCT head showing bleeding in the periventricular white matter around the right lateral ventricle with extension to the lateral ventricle NCCT: non-contrast computed tomography

He was admitted to the intensive care area and prescribed anti-hypertensives, anti-edema, and other supportive management. Electrocardiogram (ECG) showed normal sinus rhythm with tachycardia while non-contrast computed tomography (NCCT) scan of the head revealed intracerebral hemorrhage (Figure 1) with intraventricular extension. Lab investigations revealed hemoglobin of 15 gm/L, platelet count of 181 x 109/L, and total leucocyte counts of 8.36 x 109/L. The coagulation profile was deranged with an elevated active partial prothrombin time (aPTT) of 70 seconds. The rest of the blood work came normal as summarized in Table 1.

**Table 1:** Summary of the laboratory parameters of the patient during the hospital stay PCV: packed cell volume: Na: serum sodium: K: serum potassium; TLC: total leucocyte count; SGOT: serum glutamic oxaloacetic transaminase: SGPT: serum glutamic pyruvate transaminase

	Day 1	Day 3	Day 5	Day 7	Reference Range	
Hemoglobin (g/L)	154	150	152	148	130-160	
PCV (%)	47	44	46	45	40-50%	
Platelet (10^9^/L)	180	181	226	214	150-410 X10^9^	
TLC (10^9^/L)	8.36	8.71	5.97	6.27	4-10 X10^9^	
Serum Bilirubin (mg/dl)	0.6	0.5	0.8	0.8	0.2-1.0	
SGOT (IU/L)	37	21	32	41	5-40	
SGPT (IU/L)	21	26	29	24	5-35	
Albumin (g/L)	32	35	30	32	38-55	
Na (mEq/L)	138	135	138	135	135-145	
K (mEq/L)	4.7	5.1	4.9	4.9	3.5-5.5	
Urea (mg/dl)	32	28	30	29	15-45	
Creatinine (mg/dl)	1.03	0.8	0.9	0.8	0.8-1.8	
Prothrombin Time Index (%)	100	92	100	100		
Prothrombin Time (seconds)	12	13	12	14	PT (control) = 12 seconds	
Activated Partial Thromboplastin Time (seconds)	70	54	51	36	aPTT (control) = 34 seconds	
International Normalized Ratio	1.0	1.10	1.0	1.22		

His serological workup of hepatitis B and C and HIV was negative. Ultrasound whole abdomen showed normal-sized kidneys with maintained corticomedullary differentiation.

**Figure 2:**
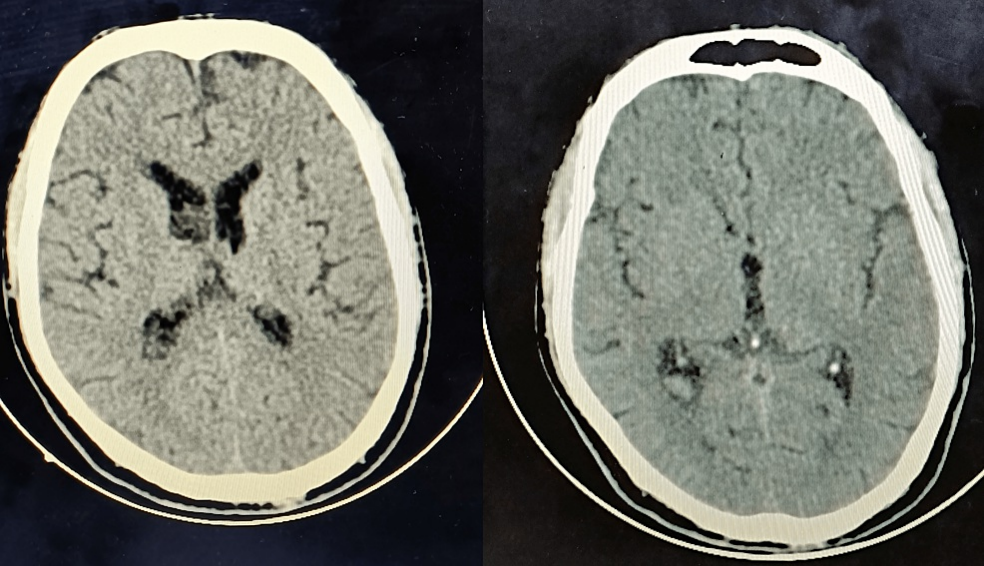
NCCT head after two weeks of the presentation showing complete resolution NCCT: non-contrast computed tomography

The patient was managed conservatively with antifibrinolytics and factor VIII (FVIII) supplementation of 1500 IU two times a day to maintain a target of 100 IU/dl. A target systolic blood pressure of 135-145 mmHg was achieved with two classes of anti-hypertensives and diuretics. A neurosurgery review was sought, and it was advised to continue monitoring the patient for raised intracranial pressure signs and symptoms. The risk vs benefits approach was taken and the decision for any operative intervention was to be consequential to deterioration in GCS. The patient’s headache resolved gradually over eight days of admission. His GCS remained unchanged and fortunately didn’t show any signs and symptoms of neurological deterioration. FVIII infusions were continued every 12 hours to maintain a residual plasma factor of 100 IU/dl. The coagulation profile was monitored regularly aimed at maintaining aPTT within normal range. A repeat NCCT head was done two weeks after the initial, which showed a complete resolution of ICH (Figure 2).

The patient was discharged with oral anti-hypertensive, supplementation of FVIII 1500 IU twice weekly with detailed information regarding red flags signs for immediate emergency consultation. He is currently following up at our tertiary care for FVIII supplementation and has been stable.

## Discussion

Despite CBDs being a recognized entity for decades and initial gene therapy trials targeting hemophilia starting as early as 1998, it still entails an incessant burden on healthcare systems worldwide. Identification of FVIII and FIX encoding genes helped in the development of recombinant clotting factor concentrates. Patients with severe HA are majorly concentrated in developing nations. The high costs of frequent infusions in severe hemophilia ~ $100,000/year make it inaccessible to the majority. Cryoprecipitate and fresh frozen plasma are still the only available measures to many in developing countries. Those born with hemophilia still die younger in the developing world with the risk being maximum in the early years of life [5].

Severe hemophilia is the most common CBD associated with recurrent bleeding complications although ICH incidence is less than in FXII D. Joint and muscle bleeding are considered hallmarks of hemophilia and the risk of ICH is documented to be high in the early days of birth. Approximately 3.5% to 4% of neonates with HA and HB experience ICH and lifelong risk remains at least 20-fold higher than that of the general population. The case fatality rate (CFR) for ICH shows a bimodal distribution, highest in age groups 0-4 years and 40-50 years. The overall incidence and mortality of hemophilia-associated ICH in all ages are 2.3 and 0.8 per 1000 person-years, respectively [1-4].

Disease severity is the most relevant risk factor in children while in adults with concomitant viral infection (e.g. HIV or HCV), anti-retroviral therapy, uncontrolled hypertension, or African American ethnicity, the development of inhibitors to exogenous infusions have been identified [1-4].

A recent retrospective review of Indian HA patients found that inhibitors increase with an increase in disease severity. About 6.7% of patients with severe HA had inhibitors in comparison to 4.2% of moderate HA patients. Inhibitors were detected at a median age of 16 years as opposed to the first decade of life documented in western studies. Atraumatic CNSB was found to be in 20% of HA patients with inhibitors vs. 4.1% of HA patients without detectable inhibitors [6,7].

Previous reviews have documented intraparenchymal and subarachnoid as two common areas involved in spontaneous CNSBs. Our patient had an intraparenchymal bleed with intraventricular extension. In the absence of a guideline regarding the end goal of therapy for hemophilia-associated ICH, it’s generally recommended to continue supplementation till radiological resolution. In spontaneous ICH, the target is to maintain 100 IU/dL for at least seven to ten days [7-10]. The risk vs. benefit that inclines management toward a conservative approach vs. a neurosurgical approach is very subtle [7]. We supplemented FVIII twice daily and monitored aPTT regularly since repeated FVIII plasma level measurements increased the financial burden significantly. The World Federation of Hemophilia (WFH) supports the use of antifibrinolytics alongside FVIII as CNSBs trigger fibrinolysis. Our patient was provided with tranexamic acid [1-4].

The recently published HAVEN 3 trial showed that emicizumab when administered once a week or once in two weeks significantly reduced bleeding rates by ~95% than those not on any prophylaxis.Emicizumab was introduced to help reduce challenges linked to multiple FVIII replacements and act as a standardized management option for HA patients with and without inhibitors. It is a bispecific monoclonal antibody that bridges activated FIX and FX to replace the function of missing activated FVIII, restoring hemostasis [11,12]. The drug was not given as prophylaxis in our case due to high suspicion of the presence of inhibitors and its unavailability, in general, due to the high financial burden.

## Conclusions

CBDs still pose a major challenge to physicians worldwide. Spontaneous ICH remains a life-threatening reality in severe hemophilia. The high cost of factor concentrates and newer emerging prophylactic monoclonal antibodies to reduce bleeding risk make the situation increasingly complicated to manage in developing nations with resource constraints. Gene therapy for hemophilia remains a distant reality in underdeveloped nations. Although the advancement has considerably increased the life expectancy of patients, a lot is required to improve disability-adjusted life years.

## References

[1] Dorgalaleh A, Farshi Y, Haeri K, Ghanbari OB, Ahmadi A (2022). Risk and management of intracerebral hemorrhage in patients with bleeding disorders. Semin Thromb Hemost.

[2] Young G (2021). Can we do something about ICH in hemophilia?. Blood.

[3] Tabibian S, Motlagh H, Naderi M, Dorgalaleh A (2018). Intracranial hemorrhage in congenital bleeding disorders. Blood Coagul Fibrinolysis.

[4] Zwagemaker AF, Gouw SC, Jansen JS (2021). Incidence and mortality rates of intracranial hemorrhage in hemophilia: a systematic review and meta-analysis. Blood.

[5] High KA (2014). Gene therapy for hemophilia: the clot thickens. Hum Gene Ther.

[6] Ray D, Kumar N, Hans C (2022). Inhibitor; an uncommon but vexing challenge in North Indian patients with hemophilia A. Indian J Hematol Blood Transfus.

[7] Aras M, Oral S (2020). Management of intracranial hemorrhage in hemophilia A patients. Childs Nerv Syst.

[8] Ghosh K, Ghosh K (2016). Management of haemophilia in developing countries: challenges and options. Indian J Hematol Blood Transfus.

[9] Zanon E, Pasca S, Demartis F (2022). Intracranial haemorrhage in haemophilia patients is still an open issue: the final results of the Italian EMO.REC Registry. J Clin Med.

[10] Hegde A, Nair R, Upadhyaya S (2016). Spontaneous intracerebral hemorrhage in hemophiliacs—a treatment dilemma. Int J Surg Case Rep.

[11] Mahlangu J, Oldenburg J, Paz-Priel I (2018). Emicizumab prophylaxis in patients who have hemophilia A without inhibitors. N Engl J Med.

[12] Mahlangu J, Iorio A, Kenet G (2022). Emicizumab state-of-the-art update. Haemophilia.

